# Emerging roles for complement in lung transplantation

**DOI:** 10.1172/JCI188346

**Published:** 2025-10-01

**Authors:** Hrishikesh S. Kulkarni, John A. Belperio, Carl Atkinson

**Affiliations:** 1 Division of Pulmonary, Critical Care and Sleep Medicine, Department of Medicine, David Geffen School of Medicine, University of California, Los Angeles, Los Angeles, USA.; 2 Department of Surgery, Comprehensive Transplant Center, Feinberg School of Medicine, Northwestern University, Chicago, Illinois, USA.

## Abstract

The complement system is an evolutionarily conserved host defense system that has evolved from invertebrates to mammals. Over time, this system has become increasingly appreciated as having effects beyond purely bacterial clearance, with clinically relevant implications in transplantation, particularly lung transplantation. For many years, complement activation in lung transplantation was largely focused on antibody-mediated injuries. However, recent studies have highlighted the importance of both canonical and noncanonical complement activation in shaping adaptive immune responses, which influence alloimmunity. These studies, together with the emergence of FDA-approved complement therapeutics and other drugs in the pipeline that function at different points of the cascade, have led to an increased interest in regulating the complement system to improve donor organ availability as well as improving both short- and long-term outcomes after lung transplantation. In this Review, we provide an overview of the when, what, and how of complement in lung transplantation, posing the questions of: when does complement activation occur, what components of the complement system are activated, and how can this activation be controlled? We conclude that complement activation occurs at multiple stages of the transplant process and that randomized controlled trials will be necessary to realize the therapeutic potential of neutralizing this activation to improve outcomes after lung transplantation.

## Challenges in outcomes in lung transplantation

Lung transplantation is currently the only life-saving therapy available for patients with irreversible, end-stage lung disease (1). Approximately 3,000–4,000 lung transplants are currently performed worldwide each year (2). One-year posttransplant survival has improved over the last three decades, yet the long-term outcomes remain dismal, with the median time to death/retransplant being approximately 6 years in the period from 2001 to 2017 (3). Multiple factors contribute to the relatively poor long-term outcomes after lung transplantation compared with transplantation of other solid organs such as the kidney. Living donor transplants are uncommon in lung transplantation, and donor variables thus affect outcomes after transplantation (4). Prior cardiopulmonary history of the recipient as well as the severity of underlying illness may result in technical challenges during surgery and require extracorporeal life support (5). Additionally, the lungs are an organ constantly exposed to infections and air pollution in the environment (6, 7). These variables can result in an exaggerated immune response in the preoperative, perioperative, and postoperative period that manifests clinically as chronic lung allograft dysfunction (CLAD).

## Complement system evolution and relevance to transplantation

**Figure 1:**
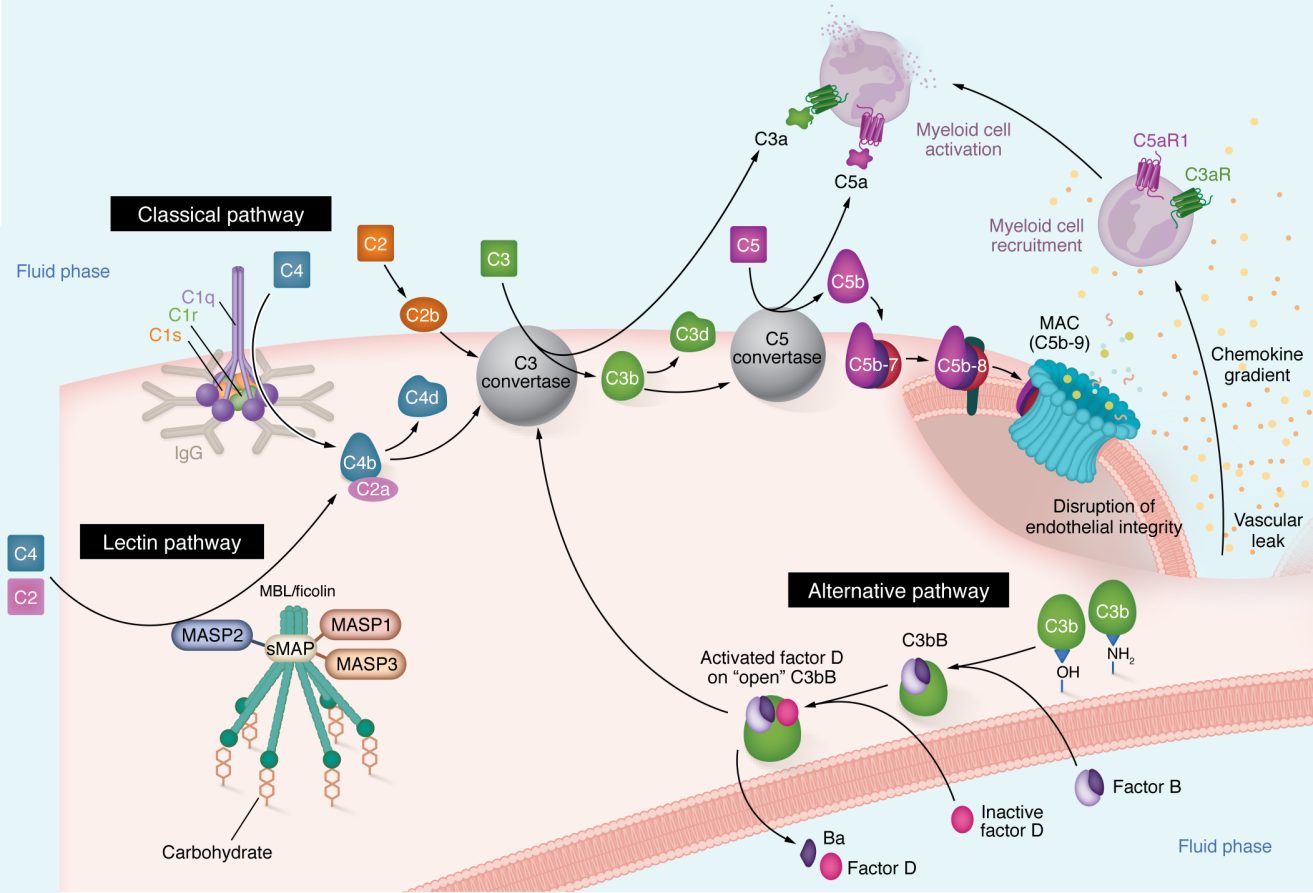
Canonical activation of the complement cascade. The complement cascade can be viewed as one that can be triggered by a series of proteases, causing it to assemble into convertases that cleave key proteins such as C3 and C5, thus amplifying the cascade and, ultimately, attacking the target. The cascade is triggered via the CP (primarily by antigen-antibody complexes) or the lectin pathway (by carbohydrates on surfaces binding to pattern recognition molecules such as mannose-binding lectin [MBL] or ficolins, and C2 and C4 cleavage by mannose-associated serine proteases [MASPs] or small MBL-associated proteins [sMAP]), to form an enzyme that cleaves C3, called the C3 convertase (C4bC2b). The alternative pathway can be initiated in the fluid phase by the conversion of C3 to C3(H_2_O), which binds to factor B, and in the presence of factor D, can generate C3b from C3. C3b binds to hydroxyl (-OH) or amine (-NH2) groups on carbohydrates or proteins on cellular surfaces via its thioester bond. Alternatively, C3b deposits directly on a surface and binds to factor B, which is then cleaved into Bb by factor D to form the (alternative pathway) C3 convertase, C3bBb. C3 convertases cleave C3 to C3a and C3b, facilitating the formation of a C5 convertase, which cleaves C5 to form C5a and C5b. C3a and C5a serve as anaphylatoxins, promoting vasodilation and chemotaxis by binding to their cognate receptors (C3aR, and C5aR1, although the role of C5aR2 continues to be clarified). C3b facilitates opsonophagocytosis and can also bind to factor B to amplify the alternative pathway. C5b binds to C6, C7, and C8 and subsequently C9 to form the membrane attack complex (MAC, C5b-9). At each step, a series of membrane regulators and fluid-phase regulators keep this system in check.

The complement system is a part of the host immune response that can be triggered to assemble, amplify, and attack as a defense against foreign cells, including pathogens (8, 9). Over time, it has evolved from invertebrates to mammals to have roles beyond bacterial clearance, including the modulation of alloimmune responses in transplantation (10, 11). The complement system comprises over 60 proteins, a majority of which are derived from the liver and present in the circulation. The system is triggered via various pathways to form a series of enzymes to amplify a cascade, release inflammatory mediators that bind to cognate receptors and form the membrane attack complex (MAC) to lyse pathogens (11). The three common pathways — the alternative pathway (AP), lectin pathway (LP), and classical pathway (CP) — facilitate enzymatic cleavage of individual complement components to converge to form C3 convertase (Figure 1), which facilitates the cleavage of C3, one of the most abundant proteins in circulation (1–2 mg/mL). Recently, granzyme K has been shown to facilitate C3 convertase formation independent of these three pathways (12). Ongoing activation and amplification facilitate C5 cleavage via both convertase-dependent and convertase-independent mechanisms and eventually lead to MAC formation (13).

**Figure 2:**
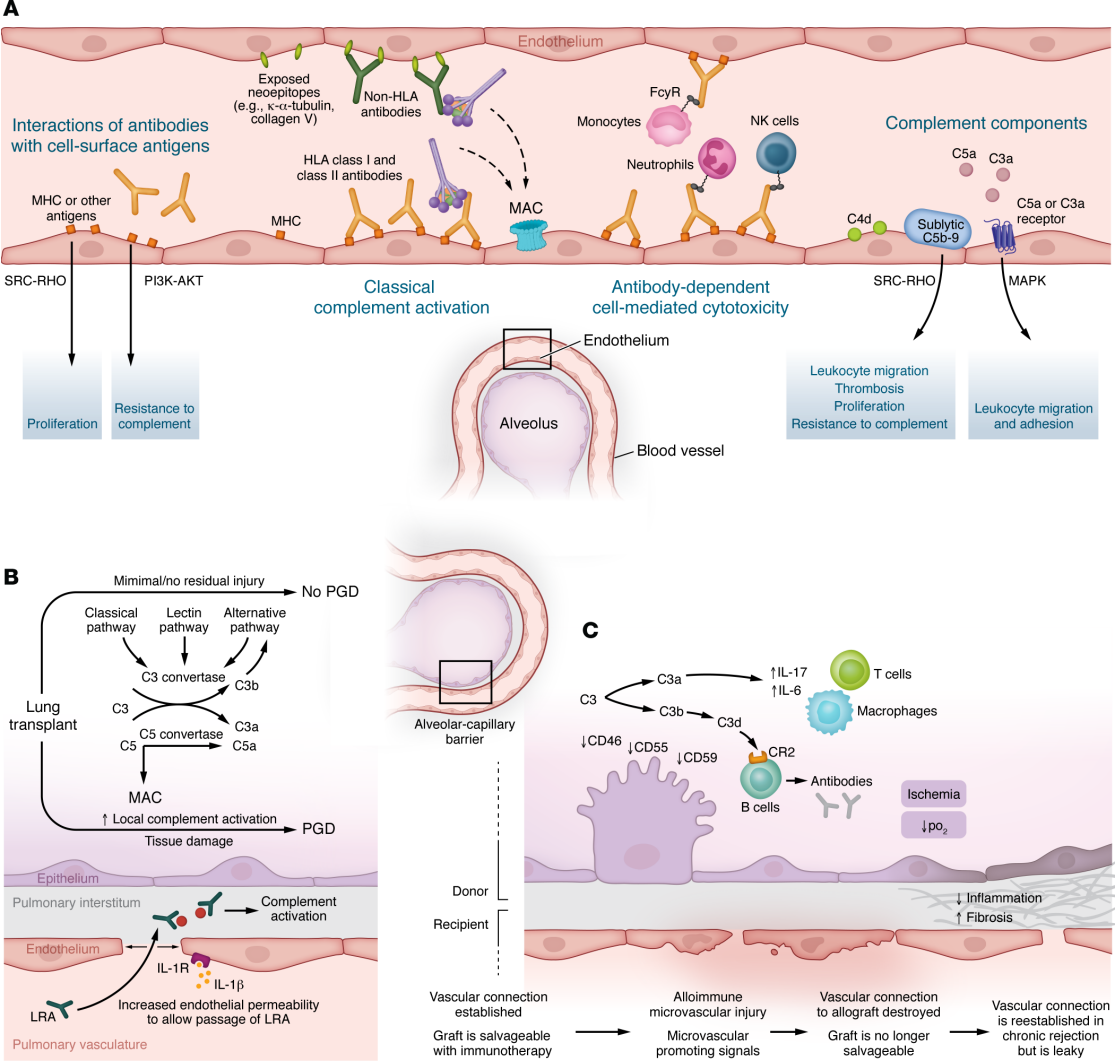
Proposed scenarios and consequences of local complement activation in lung transplantation. (**A**) Antibody-mediated rejection. Antibodies against HLA antigens and neoepitopes activate the classical complement pathway. Antibodies also activate immune cells, promoting cytotoxicity. Recent studies suggest that complement component deposition in the endothelium and signaling through the C3a and C5a receptors (especially C5aR1) can disrupt the integrity of the endothelial barrier and increase recruitment of immune cells. Additionally, signaling through noncomplement receptors can affect endothelial proliferation and thrombosis and promote resistance to complement-mediated damage. (**B**) Primary graft dysfunction. Local complement activation can damage the donor tissue, resulting in acute lung injury. Sources of complement proteins can include the tissue-resident cells, such as epithelial cells, fibroblasts, endothelial cells, and myeloid cells, but these proteins can also be sourced from the circulation in the setting of alveolar-capillary barrier disruption. (**C**) Chronic lung allograft dysfunction. Complement activation in donor lung contributes to persistent inflammation and immune dysregulation, culminating in CLAD. The effects of complement in CLAD can be attributed to the effects of activation fragments influencing B cells and/or ongoing inflammation resulting in the downregulation of regulatory proteins. For example, increased TGF-β in the lung downregulates CD46 and CD55 in the epithelium. CD59 can also be cleaved, in addition to being downregulated. MAC, membrane attack complex.

**Figure 3:**
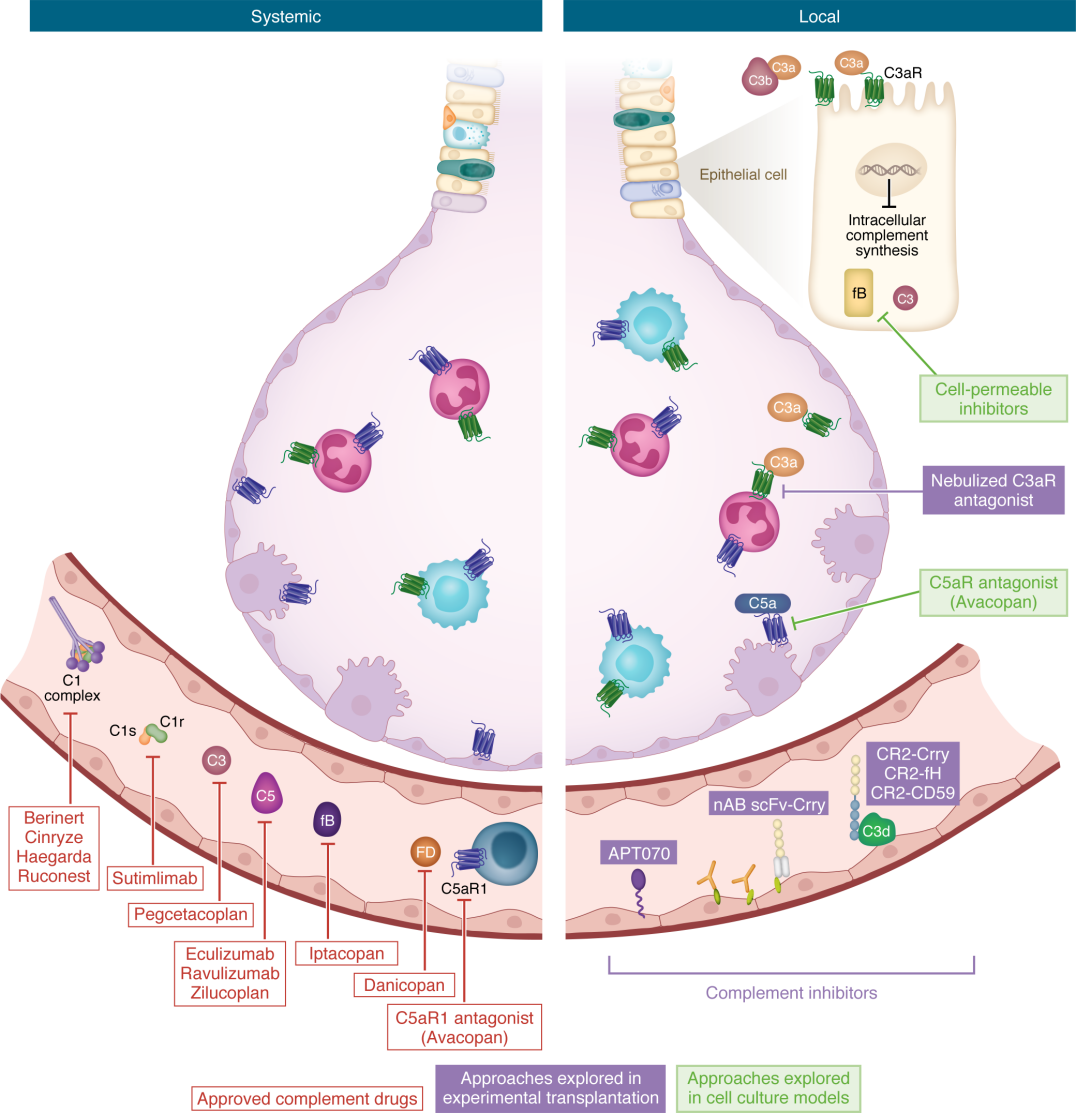
Therapies for systemic and local complement inhibition. (Left) Currently available FDA-approved complement therapeutics function via systemic inhibition of complement activation. These include inhibitors of the initiation stage (C1 esterase inhibitors [such as berinert, cinryze, haegarda, or ruconest]) or those targeting C1s (such as sutimlimab), central component C3 (pegceptacoplan), the amplification loop (iptacopan, targeting factor B; danicopan, targeting factor D), or at terminal effector pathways (eculizumab, ravulizumab, or zilucoplan, targeting C5) and C5a signaling (avacopan) (Table 3). (Right) However, given that activation of complement is a local event, there is potential to inhibit complement at the level of the graft to modulate it locally without affecting host systemic complement functions. To date, these approaches have been explored only in experimental transplantation and/or cell culture models. APT070 (mirococept), is a membrane-localizing C3 convertase inhibitor that has been explored in kidney transplantation (120). Due to its unique membrane-interacting synthetic peptide, which mediates binding to phospholipids on the cell surface, it can be perfused into the donor graft to precoat the endothelium prior to transplantation. Recombinant protein (134, 137) and natural antibody single-chain fragment (53) targeting moieties have been used to target complement inhibitors to the graft via binding to complement opsonins or exposure of damage-associated molecular patterns and/or neoantigens that are exposed by ischemia/reperfusion in the graft, respectively. Given the unique structure of the lung, direct targeting of complement can also be achieved by nebulization. Preclinical studies directly nebulizing C3aR antagonist to the donor lung have shown efficacy in reducing IRI and rejection onset (63). While not specifically tested in lung transplantation, epithelial intracellular factor B inhibition (28) and C5aR1 antagonism (33) have shown promise in reducing lung epithelial injury.

Evolutionarily, the oldest components of this system — identified in the sea anemone and horseshoe crab —are those belonging to the AP (8). The AP is activated spontaneously by hydrolysis of an internal thioester bond [when C3 undergoes conversion to C3(H_2_O)], which occurs at the rate of 1%–2% in the circulation (14). However, this rate is accelerated upon contact with various proteins, lipids, and carbohydrate structures on microorganisms and other foreign surfaces, which causes the formation of the AP convertase with a cleaved moiety of factor B (C3bBb), which can cleave C3. C3(H_2_O) generation is also accelerated in the setting of both systemic and local inflammation, including in antibody-mediated rejection (AMR) after lung transplantation (15). Subsequent to the AP, components of the CP and the LP evolved from amphibians to mammals. The CP can be triggered by the pattern recognition molecule C1q binding to antigen-antibody complexes and via antibody-independent mechanisms, and the LP can be triggered by the binding of pattern recognition moieties such as lectins to carbohydrates on the surface of pathogens and damaged cells. Activation of these pathways involves cleavage of the complement proteins C4 and C2 and results in the formation of the CP and LP convertase (C4bC2a), which, again, cleaves C3 and subsequently forms components that can form the C5 convertase and, eventually, the MAC (Figure 1). Each step of this system is tightly controlled by fluid-phase regulators in the circulation and membrane regulators on cell surfaces (16). Impaired regulation predisposes the host to autoimmunity and has been observed in models of transplant rejection (Figure 2). These regulatory proteins have also been leveraged therapeutically in xenotransplantation (Figure 3) (17).

## Cell-type-intrinsic complement production and function

It has been proposed that the complement system originated intracellularly in single-cell organisms and subsequently adapted to be secreted for host defense in multicellular organisms (18, 19). Notable observations vis-a-vis an operational intracellular complement system have supported this theory, both in immune and nonimmune cells (20–23). For example, C3 is constitutively expressed in CD4+ T cells, is required for their survival, and facilitates the skewing of a naive CD4^+^ T cell toward a Th1 phenotype (20). C3 is also expressed in innate immune cells, such as macrophages (24, 25), and nonimmune cells, such as epithelial cells, in the lung (21, 26–28), endothelial cells (29), and fibroblasts in the synovium (30) and gut (31). Moreover, C3 activation via intracellular enzymes such as cathepsins results in the generation of C3a, which can bind to an intracellular receptor (C3aR) and promotes mTOR phosphorylation (20). C3 can also bind to other ligands, such as ATG16L1 and Frk, which facilitate autophagolysosome formation and cell survival, respectively (22, 32). In addition, other components of the complement cascade have been detected intracellularly. C5 is expressed in epithelial cells and monocytes, among other cell types, and is activated by an intracellular C5 convertase to control IL-1β via mitochondrial C5aR1 signaling (25, 33). Intracellular factor B facilitates cell survival in epithelial cells, although its specific intracellular binding partner remains to be identified (27). Cellular proliferation facilitated by intracellular C1s (a protease required for CP activation), and factor H (a soluble glycoprotein that regulates AP) has been reported in certain cell types. Many of these effects are ascribed to the noncanonical roles of the complement system (34). Thus, the intracellular activity of these proteins appears to modulate cell-type-specific phenotypes relevant to disease progression and should be interrogated when these systems are targeted therapeutically. Moreover, the dynamic modulation of cell surface regulators (such as CD46, CD55, and CD59) in the setting of inflammation predisposes tissues to injury and fibrosis (35–38).

However, most of the work on intracellular complement production and activity has been done in systems other than lung transplantation. Novel tools such as complement reporter mice have enabled investigation of complement activation as a part of dynamic in vivo immune responses (20, 39). The use of C3 reporter mice has facilitated the interrogation of how cell-type-specific C3 is induced during transendothelial diapedesis via integrin lymphocyte function-associated antigen 1 (LFA-1) signaling (20). Deletion of C3 from epithelial cells in the lung has demonstrated how epithelial cell–derived C3 mitigates bacterial pneumonia–induced acute lung injury (27). C5aR1 ablation has confirmed that the C5a/C5aR1 axis in renal macrophages promotes ERK- and AKT-dependent survival during fungal infection (24). Bone marrow irradiation followed by adoptive transfer experiments have demonstrated how hematopoietic deletion of factor H affects intracellular C3 levels in macrophages and promotes lesional efferocytosis in models of atherosclerotic vascular injury (40). Reporter mice for C3aR1, C5aR1, and C5aR2 have been utilized in models of airway disease to track expression in both immune and nonimmune cells in the lungs (41–43). These models provide an opportunity to investigate cell-type-intrinsic complement production in both the donor and the recipient in models of orthotopic lung transplantation.

## The role of complement in lung transplantation

There is an increasing understanding that activation of the complement system plays key roles in the pathophysiology of organ transplant injury (Figure 2). Indeed, even before transplantation occurs, several components of the complement system have been implicated in end-stage lung diseases that require lung transplantation, such as cystic fibrosis, chronic obstructive pulmonary disease, and interstitial lung disease, as detailed elsewhere (13, 44–49). However, how the cell-type-specific expression of complement components modulates cellular responses to lung injury and repair is only beginning to be understood (21, 26, 27, 49).

The study of complement activation in organ transplantation was for many years focused on canonical effects, which include CP activation by donor-specific antibodies (DSAs) (50, 51), LP and AP activation by injury-exposed cryptic neoepitopes and necrotic debris exposed/released during ischemia/reperfusion injury (IRI) (52, 53), and LP activation by opportunistic infections (54). However, recent studies have also highlighted the importance of complement activation on shaping adaptive immune responses (55–57), which are attributed to both canonical and noncanonical effects. Canonical effects on the adaptive immune response involve activation fragments such as C3a and C5a binding to their cognate G protein–coupled receptors, C3aR and C5aR1, respectively, and promoting allograft rejection (57–60). Additionally, regulators such as CD46 and CD55 are downregulated in CLAD and are associated with increased intrapulmonary C3a levels (36). Noncanonically, complement components can influence alloimmune responses by promoting effector T cell survival and function and macrophage cytokine production and efferocytosis (20, 25, 40). In addition, local complement production in tissues affects survival and metabolism in nonimmune cells such as epithelial cells and fibroblasts (21, 30).

These studies, together with the emergence of FDA-approved complement therapeutics, which function at different points of the complement cascade (Figure 3), has led to increased interest in the role of the complement system in organ transplantation (61). FDA-approved therapeutics inhibiting C3, factor B, or C5 are likely to halt the amplification of the cascade and formation of the MAC. However, further interrogation is needed in the context of lung transplantation to determine whether inhibiting these key proteins affects graft function. It will also be critical to determine the relative importance of antibody- versus nonantibody-mediated injury in lung allograft rejection. Here, our goal is to provide an overview of the when, what, and how of complement in lung transplantation, i.e., *when does complement activation occur*, *what components of the complement pathway are activated*, and *how can this activation be controlled?*

### Donor organ injury.

Donor lungs are procured either from brain-dead (BD) or donation-after-cardiac-death (DCD) donors, each sustaining inflammatory injury before retrieval. Whether complement is locally activated within human donor lungs remains unknown; however, two murine studies show that brain death triggers robust activation of the classical and lectin complement pathways, with C3/C4 fragment deposition in pulmonary tissue and heightened injury (62, 63). Mice lacking key complement components were protected from this injury, directly implicating complement as a proximate driver of BD-induced lung damage (62). A subsequent preclinical study confirmed these findings in an independent BD model (63). Collectively, these data establish complement activation as a pivotal mediator of BD-related donor-lung injury and highlight the need to determine its role in DCD lungs, where local complement activity has yet to be examined.

### Primary graft dysfunction.

Primary graft dysfunction (PGD) occurs within the first 72 hours after transplantation. The pathophysiology of PGD is complex; however, the acute lung injury that ensues after transplant is predominantly due to IRI. Complement activation plays a key role in the propagation of ischemic injury. Binding of natural antibodies to cryptic neoepitopes that have been exposed as a consequence of ischemic insult initiates the pathogenic sequelae leading to full-blown complement activation (53, 64, 65). A study utilizing plasma samples from the NIH-funded Lung Transplant Outcomes Group demonstrated the presence of C3a, C4a, and C5a at various time points after transplantation (66). The median change in plasma C5a levels between 6 and 24 hours was significantly greater in patients who developed PGD. Furthermore, increased levels of C3a and C5a were associated with increased mortality, independent of PGD risk (66). A separate multicenter study revealed that levels of soluble C4d and C5b-9 (sC4d and sC5b-9) were significantly elevated within the first 24 hours after transplantation in the bronchoalveolar lavage (BAL) fluid of patients who developed PGD (67). Subanalysis showed that individuals with PGD also had higher C1q, C2, C4, C4b, Ba, and mannose-binding lectin (MBL), suggesting CP, LP and AP involvement. Increased levels of complement components were more evident in BAL as compared with plasma, suggesting that local lung complement activation was important in the development of PGD (67). A subsequent study demonstrated a temporal correlation between intragraft complement deposition and severe PGD. Using biopsies procured before transplant and 30 minutes after transplant, the authors demonstrated that the presence of C4d, assessed by immunostaining, was strongly associated with PGD development. Furthermore, multivariate logistic regression analysis showed that positive C4d staining was highly predictive of severe (grade 3) PGD (68). These studies demonstrate that early, persistent complement activation correlates to PGD severity.

### Acute cellular rejection.

The role of complement activation in acute cellular rejection in the lung has not been rigorously investigated. Several studies in experimental solid organ transplantation have shown that complement activation fragments can prime and promote alloimmunity (these are reviewed below). However, no studies to date have correlated complement activation with acute cellular rejection episodes clinically.

### AMR.

Drawing from experiences in other solid organ transplants, such as heart and kidney grafts, lung AMR was first diagnostically clarified in the International Society of Heart and Lung Transplant Standardization of Nomenclature in the Diagnosis of Lung Rejection, which proposed that small vessel intimitis, together with C4d and CD68 immunostaining, are potential indicators of lung AMR (69). Immunohistochemistry for C4d has been used extensively utilized as a diagnostic hallmark of AMR, with studies showing that C4d deposition correlates with septal capillary damage and necrosis (70, 71). In addition, studies have shown increased tissue deposition of C1q, C3, and C5b-9 associated with septal capillary damage (72) and circulating anti-HLA and non-HLA antibodies (73, 74). However, C4d staining has demonstrated variable, focal, and nonspecific patterns across different diagnostic groups, including acute and chronic rejection (75). While subendothelial C4d deposition may indicate HLA antibody involvement in lung allograft rejection, its patchy distribution and low sensitivity are still diagnostic concerns (76–78). As such, additional histopathological and immunohistochemical features have been investigated that correlate with probable or possible AMR, including alveolar septal widening and phosphorylated S6 ribosomal immunoreactivity (79, 80).

In addition to histopathological studies, the presence of complement activation has been assessed in BAL. Independent studies have demonstrated that sC4d levels in BAL correlate with increased circulating DSAs and biopsy-confirmed endothelial C4d deposition (81–83). A BAL approach likely offers a broader sampling field than transbronchial biopsies, which are inherently limited by their small and focal tissue yield. While increased levels of sC4d in BAL have been associated with AMR, sC4d also correlates with pulmonary infections, raising concerns that its presence may reflect generalized immune activation rather than rejection-specific pathology (81). Similarly, pilot studies have demonstrated elevations in C3(H_2_O) levels in BAL fluid prior to clinical onset of AMR (15). C3(H_2_O), a hydrolyzed form of C3 generated during complement activation, is known to rise in response to inflammation but lacks mechanistic specificity. These findings underscore the potential of complement activation products as early biomarkers of injury, but also highlight the limitations of relying on soluble factors alone to differentiate rejection from other causes of allograft inflammation.

Traditionally, AMR is defined by the presence of DSAs, histologic injury, and C4d deposition. However, as outlined above, many AMR cases, particularly in the lung, are C4d negative, lacking detectable complement activation despite clear clinical, serologic, and histologic signs of rejection (84–88). Based on these C4d-negative AMR cases and observations primarily from kidney transplantation literature, it has been proposed that there are complement-independent AMR endotypes, in which DSAs mediate injury through Fcγ receptor–dependent (FcγR-dependent) pathways, such as antibody-dependent cellular cytotoxicity, phagocytosis, and endothelial activation (86, 88–90). Within this complement-independent subset, NK cells have emerged as central effectors of graft injury (88, 89). Therapeutically, this evolving understanding opens the door to endotype-directed strategies and may enable more precise and effective immunomodulation, improving outcomes in sensitized recipients and extending long-term graft survival (88–90).

Endotyping of AMR in lung transplantation will require precise delineation of the underlying immune effector pathways active within the graft, which cannot be reliably captured by histopathology or conventional C4d staining alone (89). Achieving this granularity will depend on a combination of molecular, cellular, and imaging-based diagnostics that can resolve complement-dominant versus FcγR/NK cell–mediated injury. Complement imaging reagents represent a particularly promising avenue for noninvasively detecting ongoing complement activation within lung tissue. While current strategies have focused on opsonin-targeted probes, such as those recognizing C3d, these markers can often reflect past or inactive complement deposition and may fail to capture dynamic or sublytic activation states (91–94). A more refined approach would involve engineering imaging reagents that bind to components associated with active convertase complexes (e.g., C3/C5 convertases or their stabilizing cofactors such as properdin or factor B cleavage products). Such reagents would allow real-time visualization of functional complement activation, offering a more specific readout of active tissue injury. This could not only distinguish between complement-positive and complement-negative AMR endotypes, but also enable longitudinal monitoring of therapeutic responses to complement inhibitors, ultimately facilitating personalized, mechanism-targeted therapy.

### CLAD.

Complement activation has been increasingly associated with CLAD and its primary pathological manifestation, obliterative bronchiolitis (OB). Activation of the complement cascade contributes to persistent inflammation and immune dysregulation, which are key factors in the development of CLAD. Histopathological studies assessing C4d and C3d deposition have shown that increased levels are associated with the early onset of chronic graft dysfunction or persistent graft failure (95, 96). C1q and C4d deposition in the bronchial wall have been noted in patients with OB and have been shown to be predictive of OB (72, 96, 97). Furthermore, deposition of complement was significantly more common in patients with higher titers of anti-HLA antibodies (96). Proteomic assessment of BAL samples investigating different phenotypes of CLAD demonstrated that increased levels of C1q and C4d were associated with restrictive allograft syndrome and increased mortality (98). Assessment of LP components within plasma and BAL samples taken at 3, 6, and 12 months after transplantation revealed that elevated MBL levels were associated with poor outcomes (99). In parallel, protection against complement self-damage is regulated by membrane-bound complement regulatory proteins, which include CD46, CD55, and CD59. SNPs present within the promoter regions of these regulatory proteins can influence their transcription. In a study of 137 lung transplant donors, the presence of a CD59 SNP was associated with impaired long-term survival and a significantly higher incidence of chronic rejection (100). In keeping with the loss of complement activation control, a recent study demonstrated that a C3 polymorphism (C3R102G) that is known to result in increased complement activation through impaired C3 convertase inactivation is similarly associated with worse rates of CLAD-free survival (101). Taken together, these clinical studies point toward increased lung local complement deposition and activation as potential drivers of lung injury and CLAD development. The deliberation of when complement activation occurs during the lung transplant journey (donor brain death, postlung transplant PGD, AMR, and CLAD) is summarized in Table 1.

## Cellular sources of complement contributing to allograft injury

The sine qua non feature of complement-mediated allograft injury is covalently deposited activation fragments on the tissue surface, such as C3d and C4d (102), generally identified by immunohistochemistry or immunofluorescence. Notably, an assumption in interpreting this staining is that the components required for complement activation in the allograft originate from the circulation, which has prompted therapeutic attempts to inhibit complement activation systemically, for example, using eculizumab to block C5 cleavage (103). However, this strategy has not been successful for a few reasons. First, the drugs are often not used until later in the disease course, when the injury has likely occurred and is already severe. Second and even more concerning is the inability to assess if there is effective target engagement (for example, if complement activation is sufficiently inhibited at the site of injury). Third, it does not address the local production of complement by multiple cell types in the lung graft or by infiltrating immune cells. The availability of in vivo animal models and ex vivo human lung samples affords an opportunity to assess the local production of complement in the lung allograft and determine the effects of modulation.

### In vivo animal models.

Local graft complement activation has been shown to have a profound impact on the regulation of alloreactive T cell responses, which are critical mediators of transplant rejection. To date, all of these studies have been performed in rodents and in models other than lung transplantation (see reviews, refs. 104–106). Whether similar mechanisms explain how complement activation modulates pulmonary alloimmune responses is yet to be determined. In experimental models of kidney transplantation, epithelial and vascular sourcing of C3 affects graft survival and allograft rejection, with the epithelium being the main site of C3 expression (107). This epithelial sourcing of C3 in renal allografts also modulates IRI (108). Complement components such as C3a and C5a, generated within the graft, act on their respective receptors (C3aR and C5aR1) expressed on immune cells, enhancing alloimmune responses. The C3a/C3aR interaction on DCs is required for surface expression of MHC and costimulatory molecules on the DCs, thus facilitating effective T cell priming against alloantigens in a model of skin allograft rejection (109). Allogenic DC-derived C3a and C5a is also required for effective CD8^+^ T cell responses independent of CD4^+^ T cell help and CD40/CD154 interactions in cardiac allografts (57). To further emphasize the importance of local production/activation, donor hearts from complement regulatory protein-deficient donors were used to increase local complement activation, a feature that further exacerbated the alloimmune response and graft survival (55, 56). This local complement activation not only promoted T cell priming but was further shown to recruit antigen-presenting cells like DCs. In this setting, exacerbated complement activity also increased the ability of DCs to present alloantigens to T cells, thereby fostering robust alloreactive responses (55).

More recently, studies in murine models of orthotopic lung transplantation have demonstrated how complement activation drives humoral alloimmune responses, with deleterious outcomes. Lung-restricted autoantibodies activate both the CP and AP, resulting in PGD, while increasing C3 expression in the donor epithelial cells (51). Depletion of bronchus-associated lymphoid tissue–resident Foxp3^+^ T lymphocytes resulted in complement activation on allograft endothelial cells in arterioles, venules, and capillaries and an increase in the serum titers of donor-specific IgM antibodies, thereby triggering AMR (110). Antibody-mediated complement activation in allografts has previously been associated with microvessel loss, tissue ischemia, and fibrotic airway remodeling in orthotopic tracheal transplantation (111, 112) as well as with the recruitment and activation of alloreactive T cells in models of cardiac allograft vasculopathy (113). Canonically, alloantibodies are thought to bind and activate complement leading to cellular lysis. However, recent novel studies have shown that DSAs, in conjunction with complement activation, initiate noncanonical NF-κB signaling in graft endothelial cells (113). Importantly, this signaling is triggered not by cell lysis but through sublytic MAC deposition, which activates endothelial cells without inducing death. Sublytic MAC was shown to promote endothelial expression of NF-κB–dependent chemokines, including CXCL12 and CXCL13, which recruit alloreactive CD4^+^T cells and sustain chronic vascular inflammation (113). Activation of the MAC on allograft endothelial cells results in its noncanonical internalization as well as the assembly of NIK-dependent NLRP3 inflammasome in early endosomes and IL-1β synthesis and secretion (114). This MAC-mediated activation induces IL-15/IL-15Rα expression and augments CD8^+^ T cell infiltration and alloreactive effective memory T cells, thus potentiating chronic allograft vasculopathy (CAV) (114). Inhibition of C5, or blockade of the NF-κB–inducing kinase (NIK) pathway, reduced T cell infiltration and attenuated CAV. These findings reveal a critical role for sublytic MAC as a noncytolytic but potent activator of endothelial inflammation and highlight noncanonical NF-κB signaling as a therapeutic target that bridges alloantibody-driven complement activation with chronic T cell–mediated graft injury. Thus, this ongoing localized inflammation can lead to a positive feedback loop of complement activation, sustained T cell recruitment, and cytokine production, exacerbating graft damage and rejection risk (Figure 2).

### Human donor lungs.

Given that lungs produce certain complement proteins independent of the liver (C3 and C5, which can be cleaved to form anaphylatoxins) and that membranes can activate complement, there has been a historical concern that increased complement activation may contribute to detrimental outcomes during ex vivo lung perfusion (EVLP), especially with extended duration of perfusion. Additionally, complement activation occurs and contributes to antibody-mediated injury in ABO-incompatible lung transplantation and xenotransplantation (115, 116). C5a, measured as a proxy for complement activation, has been detected in the perfusate during EVLP. However, neither the dialysis process itself, nor the length of perfusion, resulted in increased complement activation (117). Thus, the inflammatory responses associated with prolonged dialysis in EVLP are attributed to factors other than complement activation. At the same time, EVLP may provide an avenue to modulate the increased complement activation that occurs in the lungs, especially in the setting of prolonged brain death (62, 118). Although studies involving targeted complement modulation have not been performed in lung transplantation to date, rituximab, a monoclonal antibody, has been safely administered for targeted depletion of allogenic CD20^+^ B cells in human donor lungs using EVLP (119), and mirococept, a complement inhibitor, has been utilized ex vivo in donor kidneys prior to transplantation, although an optimal effective dose needs to be identified (120). Moreover, complement inhibition in humans can be done through other means, such as enzymatic treatment of human donor lungs with FpGalNAc deacetylase and FpGalactosaminidase to increase donor lung availability (115).

## Complement therapeutics in lung transplantation

Given these findings, complement inhibition has emerged as a promising therapeutic strategy in lung transplantation to mitigate PGD, AMR, and CLAD. Numerous preclinical and clinical studies have explored systemic complement therapeutics, highlighting the critical role of complement activation in driving inflammation, IRI, and immune-mediated graft damage. Investigations using rat (121), porcine (122), sheep (123), and dog (124) models of lung transplantation have shown that targeting key components of the complement cascade, including C1 inhibitors to prevent CP activation, C3 inhibitors to block central complement activation, and C5 inhibitors to mitigate terminal pathway effects, such as MAC formation and anaphylatoxin generation, can improve early graft outcomes. These animal models have provided mechanistic insights into complement-driven injury and served as a foundation for translating findings into clinical contexts (summarized in Table 2). To this end, a number of case reports and small single-center studies have investigated complement inhibition in clinical lung transplantation (summarized in Table 3). One study examined TP10, a recombinant soluble complement receptor 1 (sCR1) that inactivates C3 and C5 convertases. In a randomized, double-blind, placebo-controlled multicenter trial involving 59 patients, those who received TP10 prior to lung allograft reperfusion showed higher rates of early extubation, the primary endpoint of the study, as compared with those in the placebo group (125). Additionally, TP10 reduced ventilator days in patients at risk for IRI and cardiopulmonary bypass injury. However, no survival benefit was observed, potentially due to the heterogeneity in complement activation, lack of sustained complement inhibition, challenges in early identification of PGD severity (the study that was performed prior to development of the International Society for Heart & Lung Transplantation PGD scoring criteria), and variable responses to complement inhibition. C1 esterase inhibitor (C1-INH), a serine protease inhibitor that regulates activation of both the complement and contact systems, has also been used in recipients with severe PGD (defined by a PaO_2_/FiO_2_ ratio <100 in the immediate postoperative period) (126, 127). C1-INH–treated patients experienced shorter ICU stays and rapid oxygenation improvement compared with traditional grade 3 PGD cohorts. Although one-year survival trends favored the C1-INH group, the difference was not statistically significant, possibly due to the sicker baseline condition of these patients or contributions from noncomplement-mediated processes. Another recent case study showed that perioperative C1-INH therapy permitted successful transplantation of highly sensitized lung transplant recipients, who were refractory to classical desensitization approaches (127).

In addition to prevention of PGD, case studies investigating complement therapeutics in AMR, primarily eculizumab and C1-INH, have been reported. Eculizumab has been used with varying success in lung transplant recipients with refractory AMR, characterized by DSAs and complement deposition. In some reports, eculizumab administration was associated with stabilization of lung function and reduced inflammation markers. In one case, treatment with eculizumab, alongside plasmapheresis and intravenous immunoglobulin (IVIG), decreased DSA levels and improved oxygenation, though long-term graft survival varied (119, 128, 129). C1-INH has also been shown to reduce complement deposition and improve graft function and patient oxygenation (130, 131). Preexisting lung-reactive antibodies (LRAs), such as antibodies against collagen V and K α tubulin, have also been shown to be independent predictors of grade 3 PGD after lung transplantation (51). In a recent study, patients with preexisting LRAs and C4d on lung biopsies taken 60 minutes after transplantation who experienced lung allograft dysfunction were treated with eculizumab, along with plasma exchange. Treatment was associated with resolution of graft dysfunction (51). While these case studies provide encouraging evidence, the variability in outcomes emphasizes the need for more robust clinical trials to determine the optimal timing, dosing, and combination strategies for complement inhibition in lung transplantation.

Complement activation is a local event, yet complement inhibition is generally applied systemically to halt complement-mediated tissue injury. Multiple cell types can generate their own complement independent of hepatic sources, and there is a growing body of literature demonstrating that donor organ–produced complement plays a role in allograft injury and alloimmune priming. For example, elegant studies demonstrated that donor C3 production was central to the development of acute kidney allograft rejection (107). In one study in rats, when lung allografts from C6-sufficient (C6+) donors were transplanted into C6+ recipients, they were rejected rapidly, exhibiting pronounced vascular infiltration, diffuse alveolar hemorrhage, and vascular endothelial injury accompanied by antibody and C3d deposition. In contrast, lung allografts transplanted between C6-deficient (C6–) donors and recipients showed significantly prolonged survival with preserved vascular integrity. C6 was used to assess the role of MAC in allograft rejection, and the origin of C6 influenced the location of injury and infiltration of immune cells. In cases where the donor provided C6 (C6+ donor to C6– recipient), increased macrophage accumulation occurred in capillaries, whereas recipient-derived C6 (C6– donor to C6+ recipient) caused macrophage accumulation primarily in pulmonary arteries (132). Given these considerations relating to the source and location of complement activation and its downstream effects, targeting the lung directly to inhibit complement locally could have potential benefits. To this end, a single pretransplant dose of nebulized C3a receptor antagonist directly to the BD donor lung pretransplant was sufficient to ameliorate IRI and delay the onset of acute rejection (63). In keeping with this lung-targeted approach, recent advances in targeted recombinant fusion protein complement inhibitors have led to the development of several complement inhibitors with potential graft-targeting properties. Recombinant complement inhibitors with targeting moieties that bind either surface-bound C3 opsonins (complement receptor 2 [CR2]) or ischemia-related cryptic neoepitopes (C2) have been shown to bind to heart (133, 134), liver (135, 136) and lungs (53) after transplant. These targeted complement inhibitors, CR2-Crry and C2-Crry, were shown to bind to the transplant grafts specifically, modulate local complement activation, and mitigate early graft injury (53, 134, 136). This approach had the additional benefit that effective inhibition of injury was provided without affecting systemic complement function, something not attainable with currently available complement inhibitory approaches (53, 133). Of note, both constructs have been humanized (137–139) and, in the case of CR2, such targeting approaches been shown to be safe in humans.

These animal studies are informative but have limitations. The complement cascade retains its core architecture across mammals to orchestrate pathogen clearance and shape adaptive immunity (140, 141), but each species custom-tunes the system through distinct species-specific membrane regulators and effector properties. In humans, CD46, CD55, and CD59 efficiently restrain convertase assembly and MAC insertion. By contrast, mice substitute the multifunctional cofactor Crry for CD46/CD55 and express two CD59 isoforms (CD59a and CD59b), conferring a markedly reduced lytic capacity and shifting the injury threshold for complement-mediated graft damage (142–145). These structural differences extend to receptor polymorphisms in C3aR and C5aR1, which alter ligand affinity and downstream signaling so that chemokine release or leukocyte recruitment quantified in rodents may not scale linearly to human physiology (146, 147). Therefore, to interrogate complement pathophysiology, investigators deploy species-matched inhibitors such as the Crry-Ig, CR2-Crry fusion protein, or BB5.1 (anti-C5), which block complement locally and permit multidose or longitudinal protocols with minimal immunogenicity (134, 148, 149). Complement-humanized murine models have recently been engineered to express human C3, or key receptors and regulators, which may provide an additional platform that maintains rodent genetics yet enables direct testing of human-specific therapeutics (150). As outlined in Table 2, both rodent and large-animal models have been employed to evaluate human complement therapeutics, including C1-INH (targeting the CP) and soluble CR1 (which inhibits C3/C5 convertases), in the setting of acute IRI. These agents exhibit partial cross-reactivity in select animal species, allowing for short-term studies that yield valuable pharmacokinetic, pharmacodynamic, and safety data directly translatable to human clinical dosing paradigms. By combining these acute translational studies with species-specific inhibitors for mechanistic exploration, and leveraging emerging humanized models where appropriate, researchers can effectively bridge interspecies differences and accelerate the development of complement-directed therapies.

While not directly related to alloimmunity, a recent report investigating SARS-CoV-2–infected lung epithelial cells demonstrates the potential of modulating intracellular complement to mediate epithelial cell inflammation (28). Using a cell-permeable inhibitor of complement factor B (CFBi), modulation of C3 activation was associated with a decrease in the IFN/JAK1/2/STAT1 pathway and NF-κB activation and a concomitant decrease in epithelial cell proinflammatory cytokine release (28). Taken together, these studies shown that modulating complement locally within the lung may protect from graft injury (summarized in Figure 3).

The growing number of FDA-approved complement therapeutics that can intercept the complement cascade may present opportunities to improve outcomes in lung transplantation. Each therapeutic offers distinct advantages and trade offs. For example, upstream blockade with C1-INHs and anti-C1s antibodies may prevent CP or LP initiation and, by extension, downstream activation of the C3 amplification loop (Figure 3). This broad inhibition is likely beneficial in the context of IRI or when DSAs or LP activation are dominant drivers of injury. However, these agents also suppress early opsonization of pathogens and clearance of cell debris, potentially increasing the risk of infection (151, 152). Since all complement pathways converge at C3, inhibition of this central component (e.g., pegcetacoplan) silences all pathways simultaneously and may offer maximal antiinflammatory benefit. Whether C3 blockade significantly increases infection risk or impairs immune surveillance in already immunosuppressed transplant recipients remains unclear and requires further study. Notably, the TP10 clinical trial in PGD showed no increase in infection risk following acute C3 inhibition, and recent clinical use of pegcetacoplan in kidney xenotransplantation helps to further assuage these concerns (125, 153). Downstream targeting of C5 or the C5a/C5aR1 axis (e.g., eculizumab, ravulizumab, avacopan) preserves upstream opsonization while blocking anaphylatoxin release and MAC formation, a balance that may effectively limit tissue injury with a more favorable safety profile. Notably, clinical experience with eculizumab in lung transplantation is expanding, including its use in presensitized recipients and in the treatment of AMR (see Table 2).

Whether these agents should be delivered systemically or directly to the lung remains an open question, as unlike other solid organs, the lung provides unique opportunities for local delivery. Intrapulmonary delivery of complement therapeutics could be performed in the donor, during EVLP, or after transplantation via nebulization, a strategy that likely obviates the risk of off-target effects seen with systemic complement inhibition, such as excessive immunosuppression. On one hand, systemic therapy may better control spillover inflammation, but organ-targeted strategies may enhance drug concentration at the site of injury, spare systemic complement activity, and enable shorter treatment duration.

## Summary

Recent advances in lung transplantation have demonstrated that complement activation contributing to allograft injury is a local event. Yet complement modulatory therapies have largely been applied systemically, often with suboptimal results. By investigating the specific components of the complement cascade and the mechanisms by which they drive alloimmune responses, understanding the time course over which complement activation occurs, identifying lung transplant recipients who are predisposed to complement-mediated allograft injury, and by discerning the relevant cellular sources of complement production in the allograft, we will be able to refine our approaches for site-specific targeting. These approaches could enable personalized, mechanism-targeted therapies tailored to the dominant effector pathways and the patient’s own infection risk profile, with the ultimate goal of improving both short- and long-term outcomes after lung transplantation.

## Figures and Tables

**Table 3:**
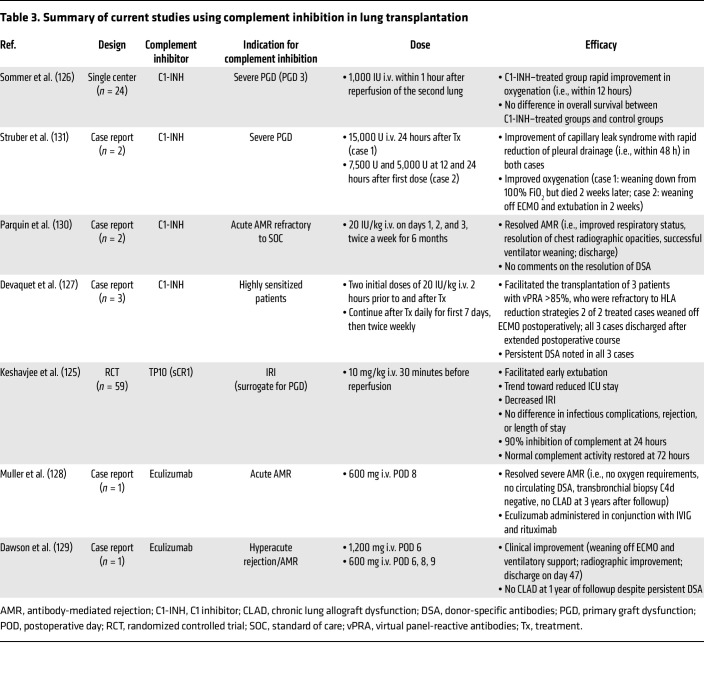
Summary of current studies using complement inhibition in lung transplantation

**Table 2:**
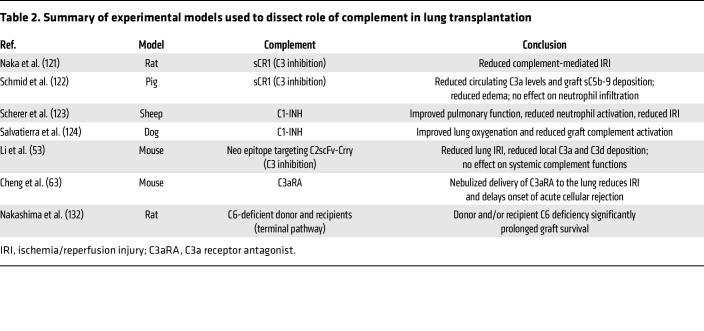
Summary of experimental models used to dissect role of complement in lung transplantation

**Table 1:**
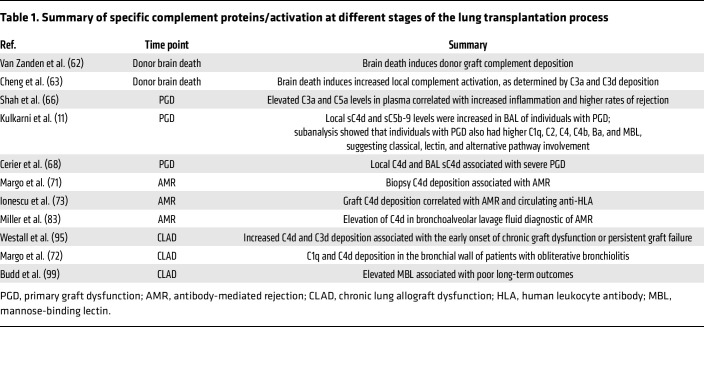
Summary of specific complement proteins/activation at different stages of the lung transplantation process

## Version 1 10/01/2025

Electronic publication
